# Genetically Determined Plasma Docosahexaenoic Acid Showed a Causal Association with Female Reproductive Longevity-Related Phenotype: A Mendelian Randomization Study

**DOI:** 10.3390/nu16234103

**Published:** 2024-11-28

**Authors:** Huajing Gao, Yuewen Ying, Jing Sun, Yun Huang, Xue Li, Dan Zhang

**Affiliations:** 1 Key Laboratory of Reproductive Genetics (Ministry of Education) and Department of Reproductive Endocrinology, Women’s Hospital, Zhejiang University School of Medicine, Hangzhou 310006, China; huajingg@zju.edu.cn (H.G.); yingyuewen@zju.edu.cn (Y.Y.); huangyun@zju.edu.cn (Y.H.); 2 Department of Big Data in Health Science, School of Public Health, Center of Clinical Big Data and Analytics of The Second Affiliated Hospital, Zhejiang University School of Medicine, Hangzhou 310006, China; sunjing1011@zju.edu.cn; 3 Zhejiang Provincial Birth Defect Control and Prevention Research Center, Hangzhou 310006, China

**Keywords:** reproductive longevity, age at natural menopause, polyunsaturated fatty acids, docosahexaenoic acid, Mendelian randomization

## Abstract

Background: Female reproductive aging remains irreversible. More evidence is needed on how polyunsaturated fatty acids (PUFAs) affect the female reproductive lifespan. Objectives: To identify and validate specific PUFAs that can influence the timing of menarche and menopause in women. Methods: We utilized a two-sample Mendelian randomization (MR) framework to evaluate the causal relationships between various PUFAs and female reproductive longevity, defined by age at menarche (AAM) and age at natural menopause (ANM). Our analyses leveraged summary statistics from four genome-wide association studies (GWASs) on the plasma concentrations of 10 plasma PUFAs, including 8866 to 121,633 European individuals and 1361 East Asian individuals. Large-scale GWASs for reproductive traits provided the genetic data of AAM and ANM from over 202,323 European females and 43,861 East Asian females. Causal effects were estimated by beta coefficients, representing, for each increase in the standard deviation (SD) of plasma PUFA concentration, the yearly increase in AAM or ANM. Replications, meta-analyses, and cross-ancestry effects were assessed to validate the inference. Conclusions: Higher plasma DHA was identified to be associated with delayed natural menopause without affecting menarche, offering a potential intervention target for extending reproductive longevity.

## 1. Introduction

The past century has witnessed a remarkable increase in human life expectancy [1]; however, female reproductive longevity, marked by two crucial time points—the onset of menstruation (age at menarche, AAM) and the cessation of natural menstrual cycles (age at natural menopause, ANM)—has still remained consistent [2]. Evolutionarily, fertility may have not adapted to the relatively rapid changes in environment and lifestyles, such as chemical pollution and nutritional excess [3,4,5,6,7,8]. Given the ongoing trend of delayed childbearing [9], identifying protective factors in daily life to extend the female reproductive lifespan has emerged as a crucial research focus.

Polyunsaturated fatty acids (PUFAs), mainly categorized into omega-3 and omega-6 families, act as bioactive lipid mediators in human health [10]. Notably, a significant decline in daily omega-3 intake, coupled with a huge increase in omega-6, has shifted the omega-6-to-omega-3 ratio from 1:1 to 20:1 today [11]. This imbalance raises concerns about whether low omega-3 and high omega-6 levels may threaten the female reproductive lifespan. Omega-3 PUFAs have been proven to be effective in preventing and treating many female reproductive diseases, such as ovarian cancer, endometriosis, and polycystic ovary syndrome, while omega-6 often has the opposite effects [12,13,14,15,16,17,18]. For reproductive longevity, several cross-sectional and cohort studies in American populations have explored the impact of omega-3 and omega-6 on the ovarian reserve, but findings remain inconsistent [19,20,21,22,23,24,25,26]. Additionally, two metabolomic studies suggested associations between fatty acids and natural menopause [27,28]. However, these studies did not account for potential confounding biases due to shared metabolic pathways of fatty acids, which may have led to false positive results, and both had relatively small sample sizes. Given that the genetic characteristics of reproductive aging exhibit significant regional variations between ethnic populations [6,29], relevant studies require a broader spectrum of ancestries and more extensive datasets. To our knowledge, this is the first study to investigate the relationship between plasma PUFAs and the female reproductive lifespan across multiple populations.

Currently, randomized controlled trials (RCTs) on this topic are still lacking, probably due to the high costs and long follow-up periods for natural menopause. Mendelian randomization (MR) offers an alternative by using random allele allocation to mimic randomized grouping within populations, providing robust causal inferences that are less susceptible to confounding [30,31]. Our study analyzed various PUFAs via MR to explore their relationships with AAM and ANM, utilizing the largest-scale data from the UK Biobank (UKB), Biobank Japan (BBJ), etc. By discussing potential confounders, we identified an independent protective factor and further replicated and assessed its impact on reproductive lifespan in both European and East Asian populations.

## 2. Materials and Methods

### 2.1. Design of MR Study

**Figure 1:**
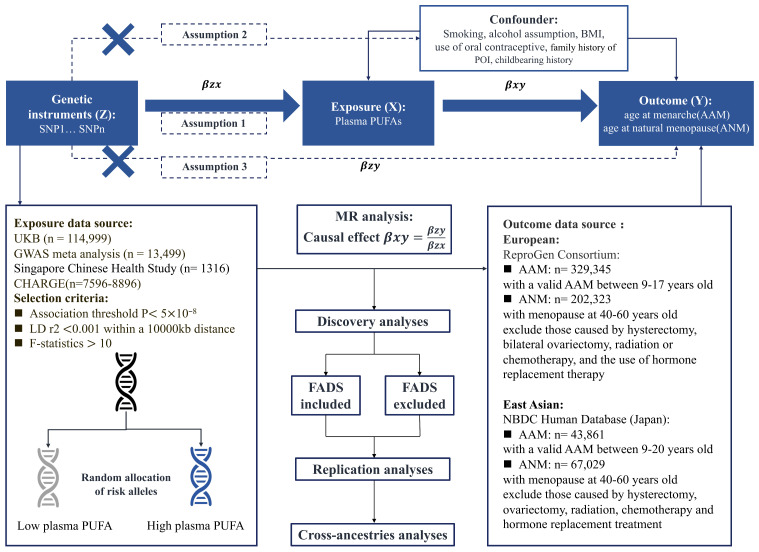
Overview of the study design and three fundamental assumptions. Abbreviations: BMI, body mass index; POI, primary ovarian insufficiency; SNP, single nucleotide polymorphism; DHA, docosahexaenoic acid; UKB, UK Biobank; n, number of individuals; GWAS, genome-wide association study; LD, linkage disequilibrium; MR, Mendelian randomization; WM, weighted median; MR-PRESSO, Mendelian randomization pleiotropy residual sum and outlier; AAM, age at menarche; ANM, age at natural menopause; NBDC, National Bioscience Database Center. FADS, fatty acid desaturase.

Figure 1 provides an overview of the study design, which followed the instruments of the STROBE-MR checklist (Table S1) [32]. We conducted a three-stage MR framework (discovery, replication, and cross-ancestry analyses) to identify the specific PUFAs causally linked to AAM and ANM. We adhered to MR assumptions: (i) instrumental variants (IVs) are strongly associated with the exposure; (ii) IVs are not linked to confounders; and (iii) IVs affect the outcomes only through exposure [33]. The first assumption was addressed through the careful selection of instrumental single nucleotide polymorphisms (SNPs). The second and third were tested through MR validation and sensitivity analyses for horizontal pleiotropy assessment.

### 2.2. Study Population and Datasets

Summary statistics for AAM and ANM in the European population were sourced from the largest reproductive genome-wide association studies (GWASs) by the Reproductive Genetics Consortium (ReproGen, an international research alliance). These studies included 329,345 Europeans for AAM and 202,323 Europeans for ANM, respectively [34,35]. AAM, defined as the onset of the first menstruation between ages 9 and 17 [34,35], was derived from self-reported questionnaires completed by women aged 40 to 60. ANM was the age at the last natural menstrual period, followed by at least 12 consecutive months of amenorrhea, excluding cases due to hysterectomy, bilateral ovariectomy, radiation or chemotherapy, or hormone replacement therapy [35].

For East Asians, AAM and ANM data were from the National Bioscience Database Center (NBDC) Human Database, based on 67,029 and 43,861 Japanese individuals in BBJ. Exclusions included menarche before the age of 9 or after the age of 20, menopause occurring before the age of 40 or after the age of 60, or medical history of hysterectomy, ovariectomy, radiation, chemotherapy, or hormone replacement treatment [36].

### 2.3. Exposure Data Source

We derived the instrumental variances (IVs) of PUFAs from four metabolomic-GWAS. In discovery stage, exposures were defined as plasma levels of five omega-3 PUFAs (i.e., total omega-3, DHA, eicosapentaenoic acid [EPA], alpha-linolenic acid [ALA], docosapentaenoic acid [DPA]) and five omega-6 PUFAs (i.e., total omega-6, linoleic acid [LA], dihomo-gamma-linolenic acid [DGLA], gamma-linolenic acid [GLA], arachidonic acid [AA]). We utilized the largest available GWAS datasets: the UKB database (over 114,999 Europeans) for plasma total omega-3, total omega-6, DHA, and LA, and cohorts for Heart and Aging Research in Genomic Epidemiology (CHARGE) consortium (7596 to 8866 Europeans) for plasma ALA, DPA, EPA, AA, DGLA, and GLA (Table S2). Those data were adjusted for age, sex, and site of recruitment [37,38,39]. For replication, we focused on plasma total omega-3 and DHA. Due to a 22% overlap between UKB and ReproGen datasets, we used another smaller GWAS of 13,544 Finnish individuals with less than 3.1% overlap, adjusted for sex and age (Table S2) [40].

For East Asian ancestry, the Singapore Chinese Health Study (SCHS) provided GWASs of plasma DHA, EPA, ALA, LA, DGLA, GLA, and AA from 1316 adult individuals, adjusted for age, sex, population stratification, and proportion of total calorie intake (Table S2) [41], with no exposure–outcome overlap.

### 2.4. Mendelian Randomization Analyses of PUFAs and Reproductive Longevity

IVs were identified at genome-wide significance (*p* < 5 × 10^−8^) and pruned by linkage disequilibrium (LD) clumping (Figure 1). Due to few SNPs meeting this threshold for DHA, EPA, and ALA in East Asian cohorts, the significance threshold for them was relaxed to *p* < 5 × 10^−6^ (Table S3). The existence of bias of weak instruments was tested through calculation of *F*-statistics with reported formulas, and SNP was removed if its *F*-statistic was <10 (calculation process can be found in Appendix A) [42].

The nearest genes of these IVs and functional annotation of SNPs, obtained from HaploReg v4.2, are listed in Table S3. Among them, human gene fatty acid desaturase (FADS), as a biomarker of PUFAs, plays a crucial role in the desaturation of fatty acids. SNPs located in the FADS region are present in the IVs for all the 10 PUFAs analyzed (Table S3).

Based on the literature and clinical experience, we identified smoking history, alcohol assumption, body mass index (BMI), history of oral contraceptive use, family history of premature ovarian insufficiency, and childbearing history as known confounders for female fertility (Figure 1) [5,7,8,29,43,44,45]. Using the Pheno-Scanner website, we excluded SNP rs2394976 (associated with former smoking, *p* = 9.7 × 10^−4^), and two BMI-related SNPs, rs112875651 (*p* = 2.94 × 10^−7^) and rs7924036 (*p* = 4.16 × 10^−10^). The remaining SNPs were independent of these confounders, and not significantly associated with outcomes.

The causality effect and the confidence intervals were calculated by inverse variance weighting (IVW) for analyses with multiple IVs or the Wald ratio for analyses with one IV [46]. We adopted a random-effects IVW model due to SNP heterogeneity, except when only two IVs were available, where a fixed-effects model was used [47].

### 2.5. Sensitivity Analyses

To eliminate the impact of potential IVs’ invalidation caused by unknown or undetectable confounders, we also performed MR-Egger regression, mode-based estimate (MBE) (including sample mode and weighted mode), and weighted median (WM) as robust validations [48,49,50]. The intercept of the MR-Egger regression was also used to evaluate the heterogeneity between different IVs as well as Cochran’s Q statistic [48]. The MR-pleiotropy residual sum and outlier (MR-PRESSO) can detect outliers and estimate results after adjusting for outliers [51]. MR-Steiger was performed for the test of directionality [52].

Fatty acids share some metabolic pathways, resulting in several common SNPs among different PUFAs. Since polymorphisms of FADS genes were identified as key genetic contributors in all the 10 plasma PUFAs of interest [53,54], we conducted causality analyses using IVs with and without SNPs from the FADS regions, respectively, to check for consistency in positive results and reduce false positives [55]. To further eliminate the potential confounding bias in replications, we excluded instrumental SNPs linked to other plasma fatty acids, including saturated fatty acids, monounsaturated fatty acids, and omega-6 and other omega-3 PUFAs. Six SNPs, namely rs139974673, rs58542926, rs77960347, rs174564, rs182611493, and rs58542926, were discovered, exhibiting a genome-wide significance level association with those (*p* < 5 × 10^−8^). Among them, rs174564 is also located in the FADS region.

All the analyses were performed in R software (version 4.2.3) with the package TwoSampleMR. Bonferroni correction modified the *p* values for multiple testing to produce a more conservative estimate of significance. *p* values < 0.005 (0.05/10) were deemed significantly associated, and those greater than 0.005 and less than 0.05 were regarded as suggestively associated. The power of the primary MR analyses was calculated on the website mRnd (https://shiny.cnsgenomics.com/mRnd/, accessed on 15 June 2024).

## 3. Results

### 3.1. Discovery Analyses Identified ANM-Related Omega-3 PUFAs

**Figure 2:**
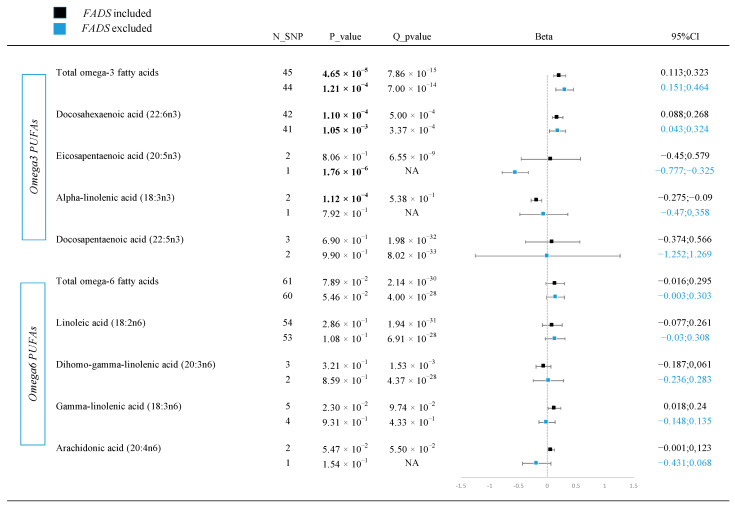
Forest plot of MR causal estimates for plasma PUFAs (five omega-3 and five omega-6 PUFAs) on age at natural menopause. Abbreviations: PUFA, polyunsaturated fatty acids; N_SNP, number of single nucleotide polymorphism; SNP, single nucleotide polymorphism; CI, confidence interval.

Genetic IVs were selected for five omega-3 PUFAs and five omega-6 PUFAs, explaining 3.7% to 30.9% of exposure variation categorized by PUFA types. Generally, both omega-3 and omega-6 exhibited a trend toward delaying ANM, with total omega-3 and DHA reaching Bonferroni-corrected significance (Figure 2). Each standard deviation (SD) increase in standardized plasma total omega-3 PUFAs concentrations was associated with a 0.218-year delay in ANM (95% CI = 0.113 to 0.323, *p* = 4.6 × 10^−5^), while DHA specifically was linked to a 0.178-year delay in ANM (95% CI = 0.088 to 0.268, *p* = 1.1 × 10^−4^). Conversely, a SD increase in standardized plasma ALA was associated with a 0.182-year earlier menopause (95% CI = −0.275 to −0.090, *p* = 1.1 × 10^−4^) (Figure 2). For age at AAM, none of the PUFAs showed significant associations, with beta estimates near zero (*p* > 1.7 × 10^−2^) (Figure S1). The study provided 100% statistical power for the Mendelian randomization analyses of PUFAs on ANM and AAM.

### 3.2. Exclusion of Potential Pleiotropic Regions Supported the Causality of Total Omega-3 and DHA on ANM

Excluding FADS regions, 1 to 60 SNPs were identified, explaining 0.4% to 4.6% of exposure variations. The exclusion did not change directions or significances of the casual inference of total omega-3 and DHA on ANM. Each SD increase in standardized plasma total omega-3 PUFA and DHA concentrations was linked to a longer extension of onset of ANM (Figure 2). Although ALA showed a causal effect on ANM in discovery analyses with FADS included, this effect was inconsistent without FADS regions (Figure 2).

After excluding FADS regions, a causal relationship emerged between the plasma AA concentration and AAM. Each SD increase in standardized plasma AA concentrations lead to a 0.247-year earlier menarche (*p* < 1 × 10^−4^). The other nine PUFAs were not associated with the AAM in analyses that both included and excluded the FADS region with a statistical power of 100% (Figure S1). In general, there is limited evidence supporting a causal relationship between PUFAs and AAM in our analyses.

### 3.3. Replication and Meta-Analyses Verified the Protective Effect of Total Omega-3 and DHA on ANM

**Figure 3:**
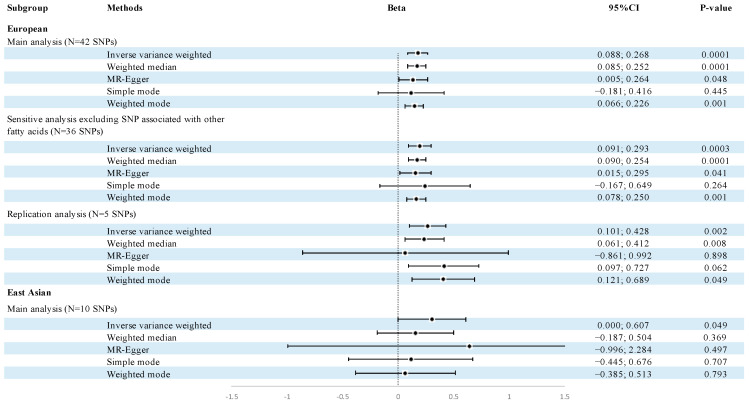
Forest plot of MR causal estimates for plasma DHA on age at natural menopause in two ancestries with replication analyses. Abbreviations: DHA, docosahexaenoic acid; CI, confidence interval.

Building on the aforementioned findings, our subsequent studies focused on the impact of total omega-3 and DHA on the ANM. In replication analysis, five SNPs were selected as IVs, explaining 2.31% of the variance in plasma total omega-3 levels, and the other five selected SNPs represented 1.95% of the variance in plasma DHA levels. Consistent with our expectations, the replication analyses yielded similar causal relationships for plasma total omega-3 PUFAs and DHA to ANM. One SD increase in standardized plasma total omega-3 was associated with a 0.241-year increase in ANM (95% CI = 0.091 to 0.391, *p* = 2 × 10^−3^), and one SD increase in standardized plasma DHA was associated with a 0.264-year increase in ANM (95% CI = 0.101 to 0.428, *p* = 1.5 × 10^−3^) (Figure 3, Table S4). The protective effect for omega-3 on ANM was confirmed by the consistent causality in both discovery and replication analyses. However, for AAM, the replication analyses still obtained no significant associations between omega-3 fatty acids and AAM (Figure S2, Table S4). Our study provided 100% power in replication MR analyses.

To further substantiate that the protective effect of omega-3 PUFA on ANM is mainly through DHA rather than other fatty acids, we identified 6 out of 42 instruments associated with fatty acid metabolic pathways in common, as mentioned in Methods. After excluding the six instruments, the associations of DHA with ANM were still significant and the effect sizes became bigger (1 SD increase in standardized plasma DHA was associated with a 0.192-year increase in ANM, *p* = 3 × 10^−4^) (Figure 3). Multiple MR methods consistently indicated the same effect direction (Figure S3a–d). The accordant direction of effects across all methods underscores the robustness of the causal estimates.

**Figure 4:**
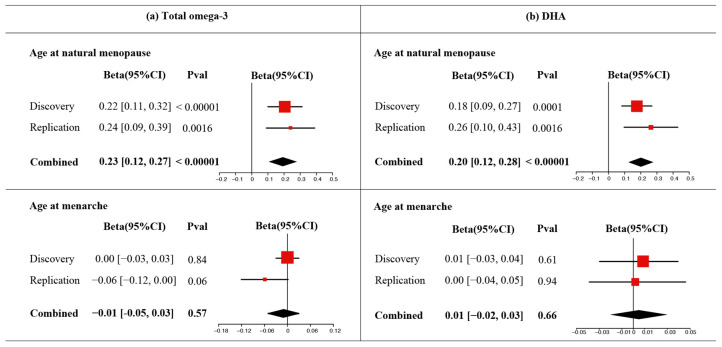
The meta-analyses of two sources of European GWASs of total omega-3 (**a**) and DHA (**b**) on ANM and AAM. The size of the red squares indicates the weight of each cohort included in the meta-analysis. The center of the black diamond represents the point estimate of the combined effect size, while the width of the diamond depicts the 95% CI. Abbreviations: ANM, age at natural menopause; AAM, age at menarche; DHA, docosahexaenoic acid; UKB, UK biobank; GWAS, genome-wide association study; CI, confidence interval.

Meta-analyses of exposure statistics from discovery and replication populations provided more convincing evidence for the protective effects of plasma total omega-3 on ANM (beta = 0.23, 95% CI = 0.12–0.27, *p* < 1 × 10^−5^) (Figure 4a) and plasma DHA on ANM (beta = 0.20, 95% CI = 0.12–0.28, *p* < 1 × 10^−5^) (Figure 4b) with no significant heterogeneity (*P*_heterogeneity_ > 0.05). In contrast, meta-analyses showed no significant associations of plasma total omega-3 or DHA with AAM (*p* > 0.05), indicating that these omega-3s do not influence the timing of menarche.

### 3.4. Sensitivity Analyses and Direction Tests Confirmed These Findings

Results of MR Steiger tests indicated that the identified significant causal relationships were not influenced by reverse causality (Table 1). The intercept values derived from MR-Egger regression were centered around zero (intercept *p* > 5 × 10^−2^), indicating a low likelihood of potential unbalanced horizontal pleiotropy (Table S4). The heterogeneity across the individual MR estimates obtained from each SNP was observed in the MR analysis of European populations (Cochran Q test *p*-value < 1.7 × 10^−2^ in both IVW and MR-Egger) (Table S4), whose impact was eliminated by choosing the random-effect type of analysis [47]. Moreover, the MR-PRESSO test found one outlier, but the association had no substantial change after removing the outlier (Table S5).

### 3.5. MR Estimates Showed Similar Results in East Asian Population

To access the effect of omega-3 and omega-6 PUFAs on ANM across ancestries, we searched GWAS datasets in other populations and found the genetic data only for East Asian available. IVs were selected for three omega-3 PUFAs (DHA, EPA, ALA) and four omega-6 PUFAs (LA, AA, GLA, DGLA) from SCHS, accounting for 0.07% to 10.7% of exposure variation across PUFA types. As expected, DHA merged as the only PUFA causally associated with ANM, with each SD increase in plasma DHA leading to a 0.303-year delay in ANM (IVW 95% CI = 0.000 to 0.607, *p* = 0.049, considered suggestively associated) (Figure 3). This association remained consistent across WM, MR-Egger, and two MBE methods (Figure S3e). For other PUFAs (EPA, ALA, LA, AA, GLA, and DGLA), no significant associations were observed, as indicated by their large *p*-values (Table S6). In the East Asian population, omega-6 PUFAs did not show a trend of delaying ANM, as the beta values were very small (Table S6). Sensitivity analyses revealed no heterogeneity or horizontal pleiotropy (Table S6), and no evidence of reverse causation was found (Table 1). Our study provided only 49% power to detect the causal effect of DHA on ANM in East Asian population, and no significant heterogeneity was found between DHA and ANM across East Asian and European populations (*P*_heterogeneity_ > 0.05). No associations were found between PUFA and AAM in the East Asian population (Figure S2, Table S6).

## 4. Discussion

Our study employed MR to analyze causal relationships between various plasma PUFA concentrations and the female reproductive lifespan, identifying DHA as a protective factor that can delay natural menopause. Replication and meta-analyses further reinforced the reliability of our findings. Sensitivity analyses eliminated potential pleiotropic effects and false positives due to fatty acid interactions and proved the absence of horizontal pleiotropy. Our studies found no evidence of plasma PUFAs affecting menarche age. Similar effects were observed in East Asian populations.

In MR analyses that included and excluded the FADS region, the specific types of PUFAs associated with ANM changed, with only DHA and total omega-3 consistently showing causal effects. Previous metabolomic studies have indicated that various fatty acids are associated with menopause or the ovarian reserve [27,28]. Since the FADS region is part of the common metabolic pathway for different fatty acids and the FADS1 gene shows strong genetic colocalization with ANM [28], FADS may influence analysis results for multiple fatty acids, potentially leading to false positives. After excluding the FADS region, DHA still showed a significant association with ANM, suggesting that DHA may be an independent protective factor for ANM. This was further confirmed by additional analyses; even after excluding six SNPs significantly associated with other fatty acids, the significant association between DHA and ANM remained, supporting the independent protective role of DHA.

Our study supports clinical applications in terms of three aspects. One contribution is filling the gap in large-scale, randomized studies. Observational studies have not consistently shown whether daily omega-3 PUFA supplementation enhances the ovarian reserve and fertility [19,20,21,22,23,24]. Although two intervention studies with 17 and 12 participants support the finding that normal-weight women may have better ovarian reserves after omega-3 supplementation, their findings require RCT validation due to small sample sizes and lack of randomization. Given the high cost and difficulty of long-term RCTs tracking natural menopause, our findings provide alternative support at the level of a large-scale population and randomized grouping. Another perspective is clarifying the effects of different omega-3 subtypes for ANM. Different omega-3 subtypes may be effective for different diseases. For instance, EPA is recommended for treating major depressive disorder while DHA is ineffective [56,57]; in hypertriglyceridemia, the use of single EPA or combination with DHA remains controversial [58,59,60]; and DHA is associated with higher sperm quality while EPA is not [61,62]. Previous clinical trials focusing on omega-3 and female reproduction mostly used fish oil supplements or combined omega-3 formulations without discussing specific subtypes. Our study was the first to suggest that single or high DHA supplements may have better clinical effects in delaying natural menopause. Finally, the fact that these PUFAs do not affect menarche is, to some extent, an advantage. Unlike the relatively stable ANM, there has been an increase in the incidence of precocious puberty in recent years [63,64], possibly due to dietary change and childhood obesity [65,66,67]. Therefore, when using nutritional supplements to extend the female reproductive lifespan, it is better to assess their impact on sexual maturation. Fortunately, our study suggests that both omega-3 and omega-6 PUFAs do not alter AAM, indicating that DHA is safe in this regard.

In addition, we explored the cross-ancestry effects and found PUFAs’ impacts on reproductive lifespan in East Asians closely mirrored those in Europeans. DHA’s effect on ANM in East Asians reached *p* = 0.049 but did not meet Bonferroni-corrected significance like in Europeans. Considering the absence of heterogeneity between the two populations, the difference likely stems from East Asians’ limited sample size (1361 individuals), yielding only 49% statistical power. Calculations indicated that a sample size of at least 2841.86 individuals would be required to achieve a power of 80%. However, the effect of DHA on delaying ANM was 1.7 times stronger in East Asians, probably suggesting greater benefits from DHA supplementation for this group.

From a biological perspective, DHA may counteract reproductive aging due to its strong anti-inflammatory properties [68,69,70,71,72]. A chronic inflammatory state is believed to accelerate ovarian senescence, resulting in compromised oocyte quality, depletion of ovarian reserves, and the development of ovarian fibrosis [73,74,75]. Omega-3 families have been proven to be a crucial anti-inflammatory role in resisting organ-specific aging [68,69,76]. Therefore, DHA’s ability to inhibit pro-inflammatory transcription factors suggests it could combat ovarian aging. Further research will explore the mechanisms and role of DHA’s anti-inflammatory effects in delaying ovarian aging.

Our study possesses several strengths. Firstly, it strictly adhered to the three principles of MR, reducing confounding bias and reverse causality. Potential confounders were discussed, and multiple sensitivity analyses confirmed the absence of horizontal pleiotropy and reverse causation. We adopted plasma PUFAs as exposures, thereby avoiding potential recall and reporting biases of self-reported dietary supplementation. Furthermore, we supplemented our study with replication research and meta-analyses. As expected, the results remained consistent in direction and significance, with *p*-values adjusted for multiple testing, greatly enhancing the reliability of conclusions while reducing the likelihood of false positives. Large-scale GWASs revealed significant differences in the genetic determinants of ANM between Asian and European populations, highlighting the enormous geographic variability and the importance of conducting studies across populations [29]. Importantly, our study is the first to examine DHA’s effects on reproductive aging in Asian populations.

Our study also has limitations. Firstly, overlapping samples between the largest GWAS databases from UKB and from ReproGen may increase type I errors and sample bias. To confirm the results, we performed a replication study with a smaller non-overlapping GWAS dataset, yielding consistent results. Secondly, we relaxed *p*-value thresholds in the SCHS GWAS for available SNPs, but F-statistics indicated no weak instrument bias. The Steiger test, which compares SNPs’ explained variances in exposures and outcomes [52], yielded very small *p*-values in the East Asian population, confirming that the IVs effectively represented the exposure. Thirdly, while our outcomes focused on women, PUFAs data were measured in both sexes, and female-specific metabolites GWASs were not available. Original GWAS data were sex-adjusted, which may partially mitigate the bias. Lastly, MR offers robust causal inferences, but further validation through RCTs is recommended.

## 5. Conclusions

This systematic MR study offers strong genetic evidence that high plasma total omega-3 and DHA levels are linked to a later age of natural menopause. DHA supplementation might be beneficial to extend reproductive longevity by delaying natural menopause.

## Figures and Tables

**Table 1:** Results of the MR Steiger test of causality for identified significant causal relationships.

Exposure	Outcome	*R*^2^ for Exposure	*R*^2^ for Outcome	Correct Causal Direction	Steiger *P*
European					
Total omega-3	ANM	4.74 × 10^−2^	5.08 × 10^−4^	TRUE	<1 × 10^−200^
Replication: total omega-3	ANM	2.31 × 10^−2^	1.15 × 10^−4^	TRUE	5.54 × 10^−58^
DHA	ANM	4.03 × 10^−2^	5.24 × 10^−4^	TRUE	<1 × 10^−200^
Replication: DHA	ANM	1.95 × 10^−2^	1.19 × 10^−4^	TRUE	4.85 × 10^−48^
Sensitivity analysis excluding SNPs associated with other fatty acids except DHA	ANM	2.29 × 10^−2^	5.07 × 10^−4^	TRUE	<1 × 10^−200^
East Asian					
DHA	ANM	1.22 × 10^−4^	8.72 × 10^−6^	TRUE	1.22 × 10^−4^

Abbreviations: ANM, age at natural menopause; DHA, docosahexaenoic acid.

## Data Availability

The GWAS data referenced in this article are available from the websites, with download links provided in Table S2.

## Supplementary Materials

The following supporting information can be downloaded at: https://www.mdpi.com/article/10.3390/nu16234103/s1, Figure S1: Forest plot of MR causal estimates for plasma PUFAs on age at menarche; Figure S2: Forest plot of MR causal estimates for plasma DHA on age at menarche; Figure S3: Scatterplots for the significant MR association between ANM and circulating omega-3 in European and East Asian populations; Table S1: STROBE-MR Checklist of Recommended Items to Address in Reports of Mendelian Randomization Studies; Table S2: Summary statistics for GWAS data of exposures and outcomes; Table S3: Genetic variants selection of plasma PUFAs; Table S4: Results of the MR causal estimates between the plasma omega-3 (SD) and female reproductive longevity; Table S5: Results of the MR analyses after removal of outliners; Table S6: Results of the MR causal estimates in East Asian population; Table S7: List of all abbreviations and their definitions.

## Appendix A

Potential bias of weak instruments was tested through calculation of *F*-statistics with the two formulas:

$$F=R^{2}(N-2)/(1-R^{2}),$$

$$R^{2}=2\mathrm{EAF}(1-\mathrm{EAF}){\mathrm{beta}}^{2},$$

where *EAF* is the effect allele frequency, *beta* is the estimated genetic effect of a given SNP on exposure, and *N* is the sample size.
