# Supplementary material for: Genetically Determined Plasma Docosahexaenoic Acid Showed a Causal Association with Female Reproductive Longevity-Related Phenotype: A Mendelian Randomization Study
Source: Nutrients. 2024 Nov 28;16(23):4103. doi: 10.3390/nu16234103 (PMC11643456; doi:10.3390/nu16234103)
Supplement: Supplementary file 1 [file nutrients-16-04103-s001.zip › nutrients-3293007-Supplementary Figures.pdf]

Supplementary Figures

■ *FADS* included
■ *FADS* excluded

Group	Exposure	N_SNP	P_value	Q_pvalue	95%CI
Omega3 PUFAs	Total omega-3 fatty acids	42	5.95×10^{-1}	5.34×10^{-18}	−0.051;0.029
		41	1.38×10^{-1}	8.79×10^{-17}	−0.107;0.015
	Alpha-linolenic acid (18:3n3)	2	5.75×10^{-1}	2.00×10^{-4}	−0.032;0.057
		1	2.19×10^{-1}	5.41×10^{-12}	−0.246;0.056
	Eicosapentaenoic acid (20:5n3)	2	2.05×10^{-1}	5.32×10^{-1}	−0.011;0.051
		1	2.85×10^{-1}	NA	−0.036;0.123
	Docosahexaenoic acid (22:6n3)	40	6.14×10^{-1}	1.54×10^{-1}	−0.025;0.043
		39	1.35×10^{-1}	NA	−0.119;0.016
	Docosapentaenoic acid (22:5n3)	3	8.89×10^{-1}	2.67×10^{-5}	−0.056;0.065
		2	4.41×10^{-1}	3.82×10^{-4}	−0.080;0.183
Omega6 PUFAs	Total omega-6 fatty acids	54	4.05×10^{-1}	2.39×10^{-15}	−0.071;0.029
		53	4.39×10^{-1}	2.15×10^{-15}	−0.070;0.03
	Arachidonic acid (20:4n6)	2	9.41×10^{-1}	1.10×10^{-10}	−0.058;0.062
		1	5.56×10^{-8}	7.47×10^{-11}	−0.336;−0.158
	Dihomo-gamma-linolenic acid (20:3n6)	3	5.39×10^{-1}	1.70×10^{-6}	−0.044;0.084
		2	1.42×10^{-1}	1.59×10^{-3}	−0.026;0.18
	Gamma-linolenic acid (18:3n6)	5	2.46×10^{-1}	3.37×10^{-5}	−0.028;0.109
		4	1.37×10^{-1}	2.44×10^{-4}	−0.030;0.221
	Linoleic acid (18:2n6)	48	3.10×10^{-1}	3.32×10^{-8}	−0.072;0.023
		47	3.91×10^{-1}	NA	−0.072;0.028

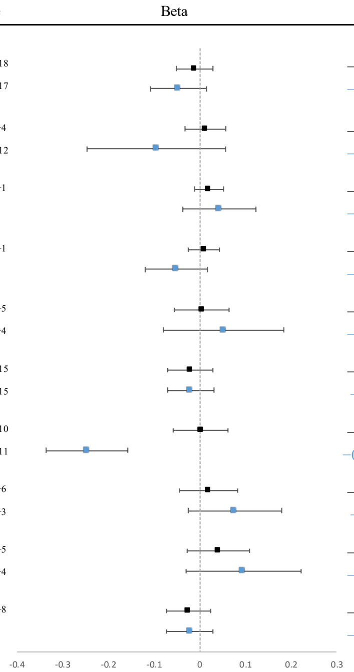

Supplementary Figure S1. Forest plot of MR causal estimates for plasma PUFAs on age at menarche. Abbreviations: PUFA, polyunsaturated fatty acids; N, number of single nucleotide polymorphisms; SNP, single nucleotide polymorphism; CI, confidence interval.

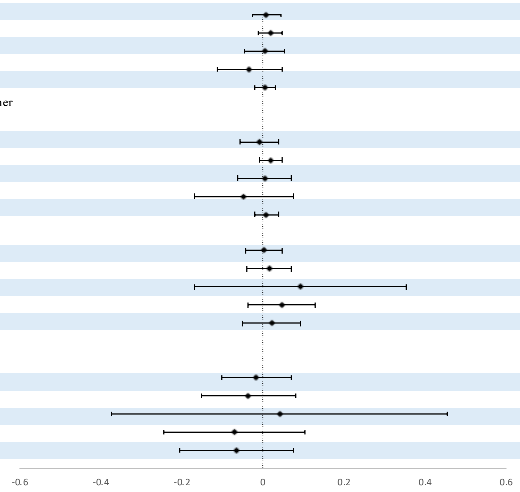

Subgroup	Methods	Beta	95%CI	P-value
**European**				
Main analysis (N=38 SNPs)				
	Inverse variance weighted		-0.025; 0.043	0.614
	Weighted median		-0.012; 0.048	0.236
	MR-Egger		-0.045; 0.052	0.881
	Simple mode		-0.113; 0.048	0.433
	Weighted mode		-0.021; 0.031	0.706
Sensitive analysis excluding SNP associated with other fatty acids (N=35 SNPs)				
	Inverse variance weighted		-0.057; 0.038	0.702
	Weighted median		-0.010; 0.046	0.225
	MR-Egger		-0.061; 0.071	0.880
	Simple mode		-0.170; 0.076	0.467
	Weighted mode		-0.021; 0.038	0.571
Replication analysis (N=5 SNPs)				
	Inverse variance weighted		-0.042; 0.046	0.939
	Weighted median		-0.039; 0.070	0.569
	MR-Egger		-0.169; 0.352	0.527
	Simple mode		-0.038; 0.129	0.331
	Weighted mode		-0.051; 0.093	0.597
**East Asian**				
Main analysis (N=10 SNPs)				
	Inverse variance weighted		-0.101; 0.069	0.716
	Weighted median		-0.153; 0.081	0.546
	MR-Egger		-0.373; 0.455	0.858
	Simple mode		-0.246; 0.103	0.467
	Weighted mode		-0.206; 0.074	0.408

Supplementary Figure S2. Forest plot of MR causal estimates for plasma DHA on age at menarche. Abbreviations: DHA, docosahexaenoic acid; N, number of single nucleotide polymorphisms; CI, confidence interval.

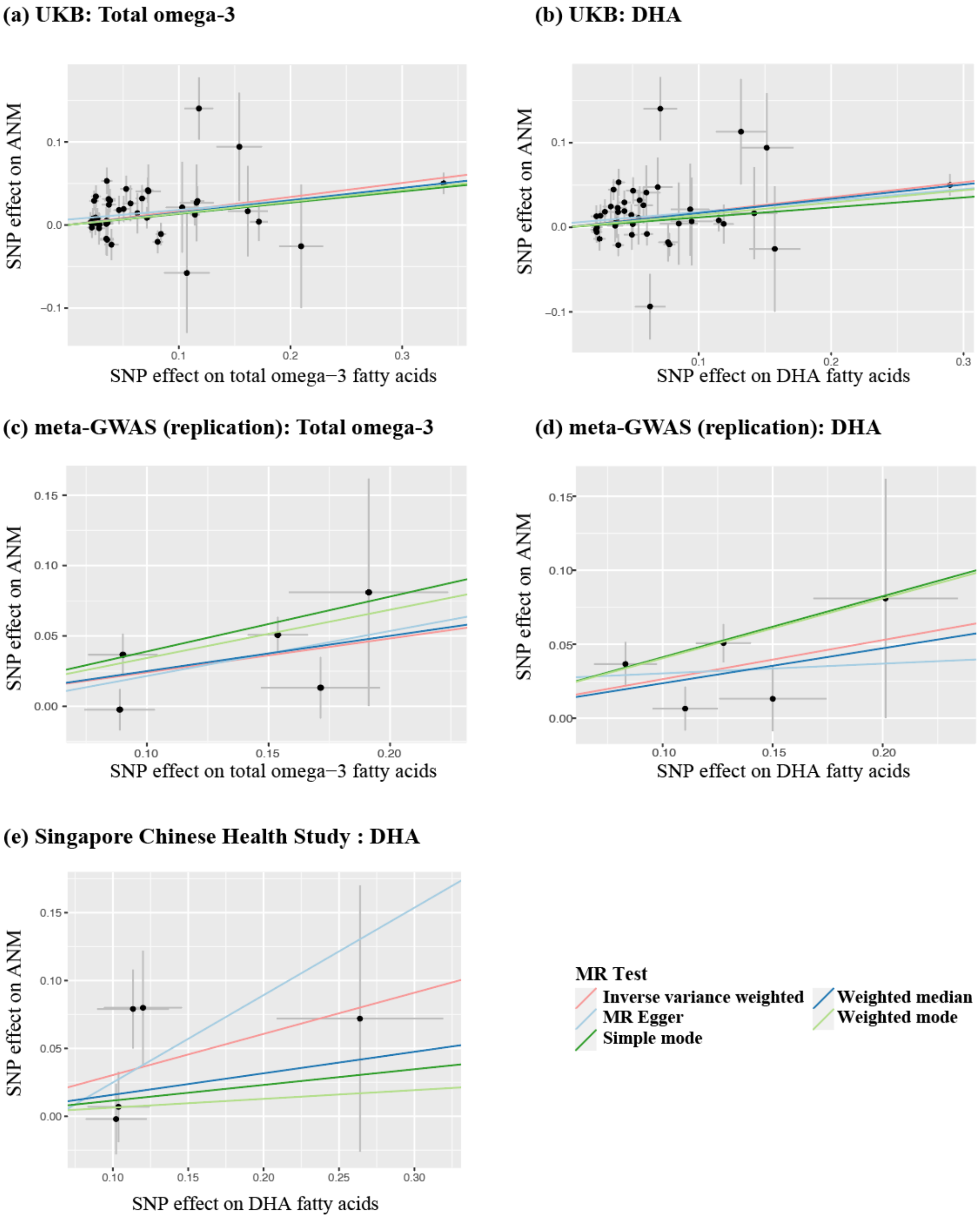

Supplementary Figure S3. Scatterplots for the significant MR association between ANM and circulating omega-3 in European and East Asian populations. Abbreviations: UKB, UK Biobank; SCHS, Singapore Chinese Health Study; ANM, age at natural menopause; SNP, single nucleotide polymorphism; DHA, docosahexaenoic acid; GWAS, genome-wide association study.
